# Spatially precise visual gain control mediated by a cholinergic circuit in the midbrain attention network

**DOI:** 10.1038/ncomms13472

**Published:** 2016-11-17

**Authors:** Ali Asadollahi, Eric I. Knudsen

**Affiliations:** 1 Department of Neurobiology, Stanford University School of Medicine, Stanford, California 94305, USA; 2 Visuo-Motor Laboratory, Rayan Center for Neuroscience and Behavior, Ferdowsi University of Mashhad, Mashhad 9177948974, Iran; 3 Department of Biology, Faculty of Science, Ferdowsi University of Mashhad, Mashhad 9177948974, Iran

## Abstract

A primary function of the midbrain stimulus selection network is to compute the highest-priority location for attention and gaze. Here we report the contribution of a specific cholinergic circuit to this computation. We functionally disconnected the tegmental cholinergic nucleus isthmi pars parvocellularis (Ipc) from the optic tectum (OT) in barn owls by reversibly blocking excitatory transmission in the Ipc. Focal blockade in the Ipc decreases the gain and spatial discrimination of OT units specifically for the locations represented by the visual receptive fields (VRFs) of the disconnected Ipc units, and causes OT VRFs to shift away from that location. The results demonstrate mechanisms by which this cholinergic circuit controls bottom-up stimulus competition and by which top-down signals can bias this competition, and they establish causal linkages between a particular circuit, gain control and dynamic shifts of VRFs. This circuit may perform the same function in all vertebrate species.

## Abstract (web summary)

Attention and gaze impact the spatial responsiveness of neurons in the optic tectum. Here the authors elucidate the mechanism by which cholinergic inputs affect receptive field properties of tectal neurons in a spatially precise manner in barn owls.

## Graphical abstract

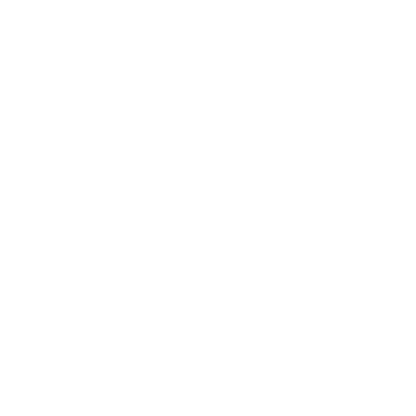

Attention-related and gaze-control signals are able to dynamically regulate the responsiveness of high-order neurons to particular stimuli or locations within their receptive fields in the primate neocortex^1^,^2^,^3^,^4^,^5^. This dynamic regulation is thought to be a critical component of attention. In this study, we demonstrate how a specific cholinergic circuit in the midbrain stimulus selection network of barn owls mediates such an effect.

Spatial attention is controlled by the coordinated interaction of midbrain and forebrain networks^6^. The midbrain network, consisting of the optic tectum (OT; superior colliculus in mammals) and several interconnected tegmental nuclei, computes the highest-priority location for attention after combining information about the relative physical salience of stimuli with endogenous information about the behavioural importance of each location^7^,^8^.

Each component of the midbrain network contains a multimodal, topographic map of space^9^. The nucleus isthmi pars parvocellularis (Ipc; analogous to a portion of the nucleus parabigeminus in mammals^10^) is a cholinergic tegmental nucleus that connects reciprocally and topographically with the space map in the OT^11^. Ipc neurons receive excitatory drive from a special class of neurons in the OT that transforms sensory input into spikes at gamma frequencies (25–60 Hz)^12^. Ipc neurons convert these periodic spikes into bursts of spikes that are transmitted back to the OT, to precisely the location that provided the input, and induce large-amplitude gamma oscillations in the OT local field potential at that location^13^. The activity of neurons in the OT that project to high-order thalamic nuclei, as well as the activity of neurons in high-order forebrain areas themselves, synchronizes with the timing of the Ipc bursts^14^,^15^.

Beyond inducing rhythmicity in the representation of the highest-priority location, how does this cholinergic circuit affect information processing in the midbrain network? We addressed this question by studying visual responses in the deep layers of the OT (which provide ascending input to high-order thalamic nuclei and descending drive to brainstem movement generators^16^,^17^) before and after blockade of excitatory transmission in the Ipc. Two properties of the Ipc allowed us to manipulate a specific class of cholinergic neurons that provide input to a particular location in the OT space map. First, the Ipc consists of a pharmacologically and structurally homogeneous population of neurons that is anatomically segregated from populations of GABAergic, glutamatergic and other classes of cholinergic neurons in the avian midbrain tegmentum^11^. Second, Ipc neurons are organized topographically in a map of space^18^. Therefore, by focally blocking excitatory, glutamatergic transmission in the Ipc space map, we were able to observe the contribution of this cholinergic circuit to information processing in the network.

Dynamic shifts in visual and auditory receptive fields have been reported previously in the barn owl OT in response to focal microstimulation of the forebrain gaze field^19^. The forebrain gaze field projects directly to the Ipc as well as to the OT^20^. Ipc neurons, in turn, project to the OT with high spatial precision^11^. Thus, the Ipc circuit provides an anatomical architecture that could support shifts of spatial tuning in the OT. Does the Ipc actually perform this function?

By applying focal blockade in the Ipc, we show that the Ipc modulates the visual representation in the OT in a spatially precise fashion. We found that Ipc blockade decreases the gain and spatial discrimination of OT units specifically for the location represented by the Ipc, and it causes OT visual receptive fields (VRFs) near the edges of the representation to shift away from this location. These effects bias the computation of the highest-priority location by the network, and they can account for the reported dynamic influences of top-down signals on the representation of space in the OT.

## Results

### Effects of the Ipc on OT spatial tuning at aligned sites

**Figure 1:**
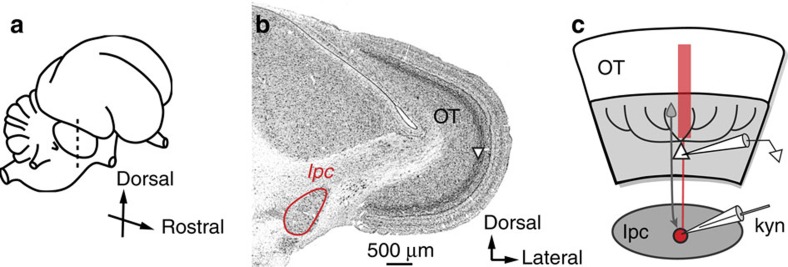
Experimental set-up. (**a**) The drawing depicts the owl brain and plane of section. (**b**) Nissl-stained, transverse section of midbrain showing the OT and nucleus Ipc (outlined in red). Layer 10 in the OT is indicated by the triangle. (**c**) Schematic of the experimental set-up: extracellular recording electrode in layers 11–13 and multi-barrelled iontophoretic and recording electrode in the Ipc. The drawing represents an ‘aligned' pair of OT and Ipc sites. Red: cholinergic neuron; shaded portion of OT: deep (multimodal and motor) layers.

**Figure 2:**
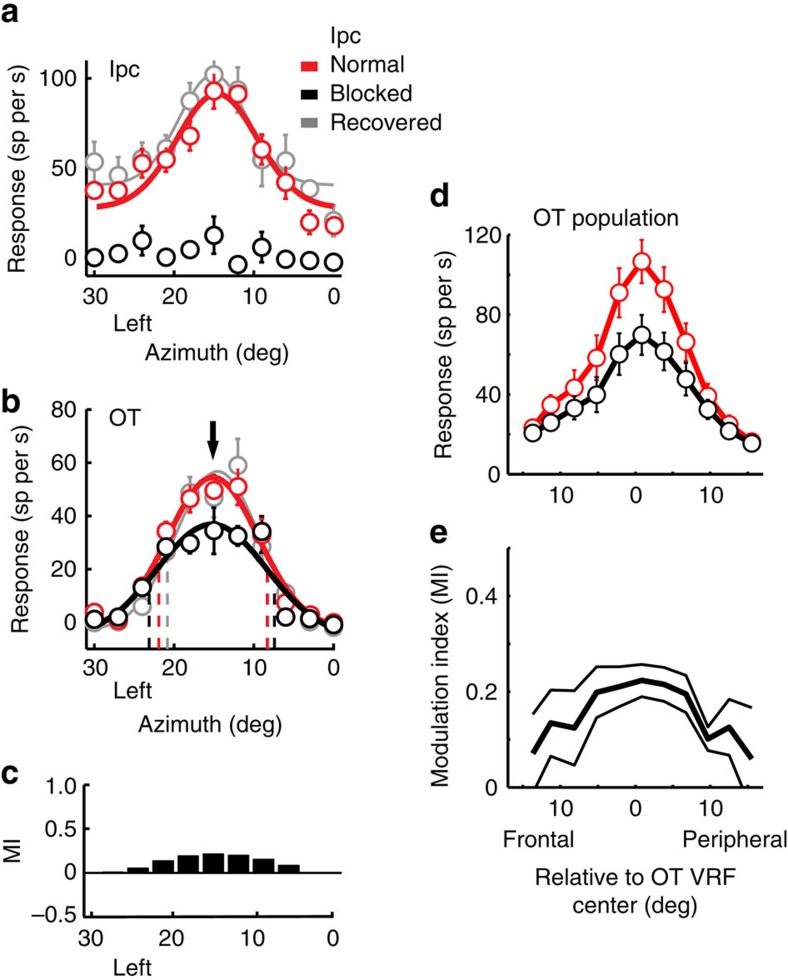
Effect of Ipc blockade on spatial tuning at aligned sites in the Ipc and OT. (**a**) Data from a multi-unit recording at the Ipc injection site. (**b**) Data recorded simultaneously from a single OT unit at an aligned site in the OT. Responses to a dark looming dot (full contrast; 8° per s) at different locations across the visual field were measured before, during and after iontophoretic application of kyn at the Ipc site. Symbols represent mean and s.e. (*n*=12); dashed vertical lines indicate locations of half-max responses; curves are best-fit Gaussian functions. Downward arrow indicates the Ipc VRF centre, measured in **a**. (**c**) Response modulation index (MI; Methods) calculated from the Gaussian fits for the responses shown in **b**. (**d**) Population average and s.e.m. of the responses of OT units from aligned OT–Ipc pairs (*n*=28) tested as described in **b**. The data are plotted in degrees frontal versus peripheral relative to the VRF centre. (**e**) Population average MI and s.e.m. resulting from Ipc blockade for OT units from aligned OT–Ipc pairs (*n*=28).

We tested the effects of blocking glutamatergic excitatory drive to Ipc neurons on visually driven responses of neurons in the deep layers (layers 11–13) of the owl OT (Fig. 1). Iontophoretic application of kynurenic acid (kyn), a broad-spectrum blocker of ionotropic glutamate receptors, reliably blocked visually driven activity in the Ipc. Figure 2a shows the effect of applying kyn at a single site in the Ipc. Before blockade, the site responded vigorously to a visual stimulus (negative contrast dot looming at 8.0° per s) located at left (L) 15°, −5° (Fig. 2a, red). During blockade, the same stimulus at the same location evoked little, if any, response (Fig. 2a, black). Across a population of such Ipc sites (*n*=28), glutamate receptor blockade reduced visually driven responses in the Ipc by an average of 56% for stimuli positioned at the VRF centre.

Single units in the OT were recorded simultaneously with the unit recordings and response blockade in the Ipc. Blockade of visually driven activity in the Ipc decreased OT unit responses, specifically to stimuli positioned at the locations represented by the units at the Ipc injection site. Figure 2b shows the reduction in responses of an OT unit that resulted from the Ipc inactivation shown in Fig. 2a. For this OT–Ipc pair of sites, both of the VRFs were centred at L15°, −5°. The responses of the OT unit decreased (analysis of variance (ANOVA); *P*<0.05) with Ipc blockade when stimuli were located near the centre of its VRF (Fig. 2b, black), locations that also corresponded to the centre of the Ipc VRF (Fig. 2b, downward arrow). The modulation of this unit, as quantified by the modulation index (Methods), peaked when the stimulus was centred in the VRF of the Ipc injection site (Fig. 2c). Responses to stimuli located >4° from the VRF centre did not decrease (ANOVA; *n*>12 repetitions; *P*>0.05). As a result, the borders of the OT unit's VRF were not altered, but the locations of the half-max response shifted away from the VRF centre (Fig. 2b, black dashed lines). In addition, the maximum spatial discriminability (max *d′*; Methods) provided by the OT unit's responses to stimuli positioned on the flanks of the tuning curve decreased (from 4.7 to 4.3). Following cessation of drug application, the spatial tuning of the OT unit returned to its pre-drug values (Fig. 2b, grey).

**Figure 3:**
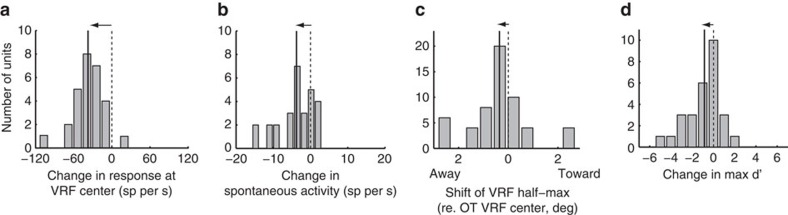
Effect of Ipc blockade on unit responses at aligned OT sites. The data compare single-unit responses in the OT measured before versus during Ipc blockade, for aligned OT–Ipc pairs. (**a**) Change in response rate measured at the VRF centre (*n*=28). (**b**) Change in the spontaneous rate (*n*=28). (**c**) Shift in the half-max locations of the VRF (*n*=56). (**d**) Change in spatial resolution, measured as max *d*′ (*n*=28). Solid vertical line: mean value; horizontal arrow indicates significant shift from zero (*P*<0.05; paired *t*-test).

Similar results were observed for the 28 pairs of OT–Ipc sites, for which OT and Ipc VRF centres were mutually aligned to ≤4°, referred to as ‘aligned pairs' (illustrated in Fig. 1c). Across this population of OT units, glutamatergic blockade in the Ipc reduced unit responses to stimuli positioned at the aligned OT VRF centre by an average of 34% (mean pre-blockade=107 spikes per second (sp per s)±11; mean during blockade=70 sp per s±10; *P*<0.001; paired *t*-test, *n*=28; Figs 2d and 3a). Ipc blockade differentially decreased responses near the centre of OT VRFs (Fig. 2d); the average modulation index for the VRF centre was 0.22±0.03 (Fig. 2e). Spontaneous activity was reduced by 27% (mean pre-blockade=15 sp per s±3; mean during blockade=11 sp per s±2; *P*<0.001; paired *t*-test; Fig. 3b). The half-max locations moved away from the VRF centres (mean shift=0.4°±0.05; *P*=0.03; paired *t*-test, *n*=56; Fig. 3c), reflecting the decrease in responses specifically to stimuli near the VRF centre. In addition, Ipc blockade decreased the maximum spatial discriminability provided by unit responses from an average max *d*′ value of 4.77±0.49–3.90±0.49 (*P*=0.01; paired *t*-test; Fig. 3d). After cessation of drug application, all response parameters returned to pre-drug values (*t*-test; *n*=28; *P*>0.1).

### Effects of the Ipc on OT spatial tuning at non-aligned sites

To study the effects of Ipc blockade on OT responses near the edges of the zone that was affected by the blockade, we repeated the same experiment on OT–Ipc pairs with differing magnitudes of VRF non-alignment. This experiment also tested for the possibility that Ipc input engages lateral inhibitory circuits in the OT. We hypothesized that if Ipc input activates lateral inhibition in the OT, then blockade of Ipc input should release non-aligned OT sites from inhibition, causing them to increase responses to locations just beyond those represented by the Ipc site.

**Figure 4:**
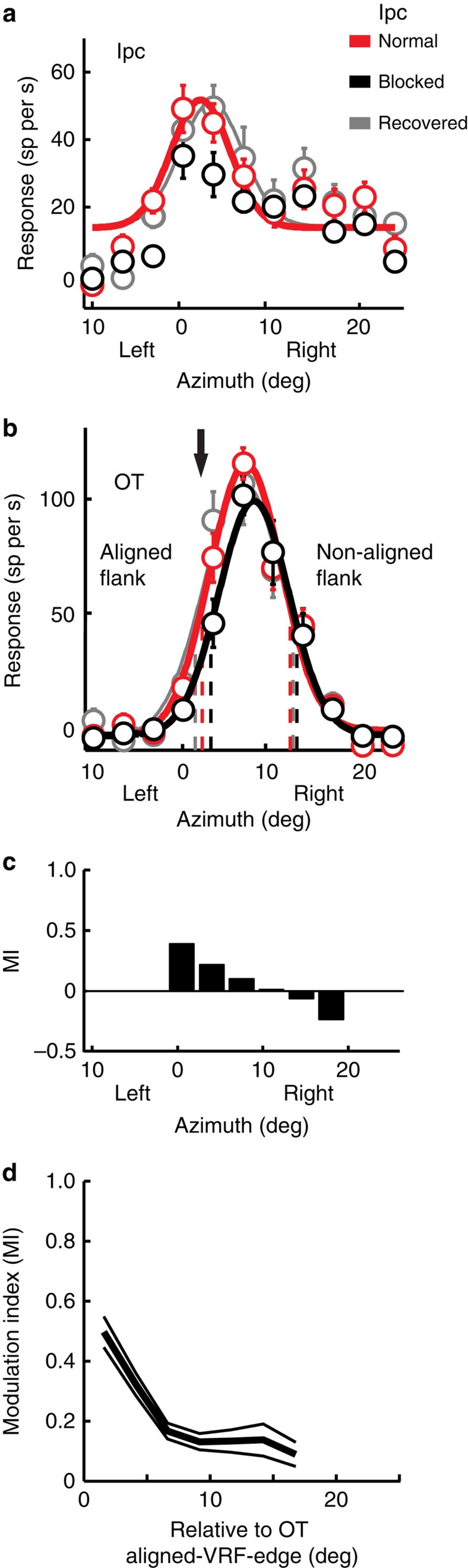
Effect of Ipc blockade on spatial tuning at non-aligned sites in the OT. (**a**) Data from a multi-unit recording at the Ipc injection site. (**b**) Data from a simultaneously recorded single OT unit at a non-aligned site in the OT. Conventions are the same as in Fig. 2. (**c**) Response modulation index calculated from the Gaussian fits shown in **b**. (**d**) Population average modulation index (MI) and s.e.m. resulting from Ipc blockade for OT units from non-aligned pairs (*n*=19). For each location, MI values were calculated only for those OT units that gave a significant response before blockade (Methods). The data are plotted relative to the Ipc-aligned border of the OT VRF (Methods).

An example of the effects of glutamatergic blockade at an Ipc site with a VRF that was located on the flank of an OT unit VRF is shown in Fig. 4. The Ipc VRF was centred at R3°, 0° (Fig. 4a), and the OT unit VRF was centred at R8°, 0° (Fig. 4b). Application of kyn, which substantially reduced responses at the Ipc site (Fig. 4a, black), decreased the responses of the OT unit for stimuli located on the flank of the OT VRF that was aligned with the Ipc VRF (aligned flank) (Fig. 4b). The largest response modulation (Fig. 4c) was for locations that corresponded to the VRF at the Ipc blockade site (Fig. 4a). As a result, the half-max value for the aligned flank moved towards the OT VRF centre and away from the Ipc VRF (Fig. 4b, black). In contrast, responses to stimuli located on the right, non-aligned flank of the OT VRF were unchanged, and the half-max value for this flank shifted slightly away from the VRF centre (Fig. 4b, black dashed line). Consequently, the spatial tuning of the OT unit shifted away from the location represented by the Ipc site (Fig. 4a).

**Figure 5:**
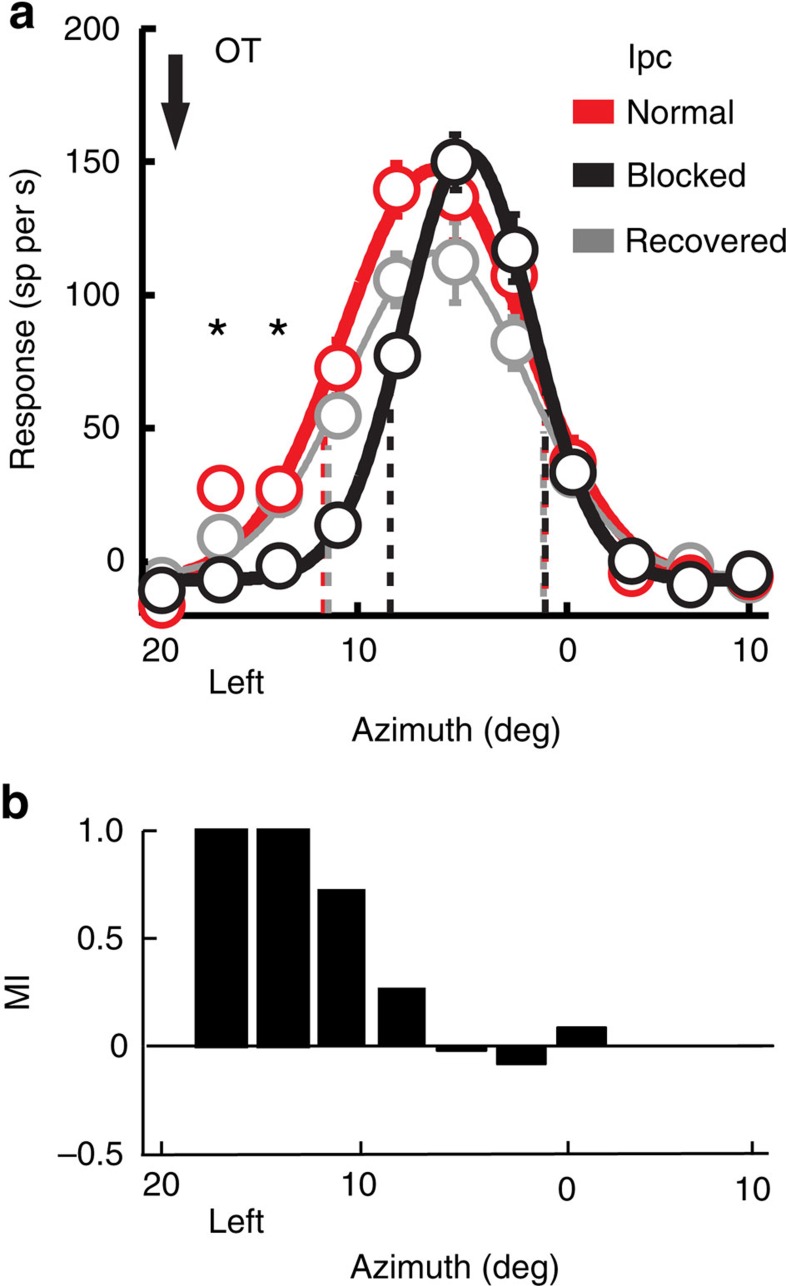
Effect of Ipc blockade on response modulation at non-aligned sites in the OT. (**a**) Data from a single OT unit for a non-aligned OT-Ipc pair. VRF locations (azimuth, elevation): OT (L6, −6); Ipc (L19, −5). Downward arrow: location of the Ipc VRF centre. Asterisks: stimulus locations that did not drive the OT unit during Ipc blockade. Conventions are the same as in Fig. 2. (**b**) Response modulation index calculated from the Gaussian fits shown in **a**. The modulation index (MI) was calculated only for those locations that yielded a significant response before blockade.

The results from 19 such simultaneously recorded OT–Ipc pairs, with various degrees of VRF non-alignment, were consistent with these observations. Ipc blockade caused the spatial tuning of OT units to shift away from the location represented by the Ipc injection sites, due primarily to a decrease in responses to stimuli located on the Ipc-aligned flank. The strength of modulation increased as the stimulus location approached the centre of the VRF at the Ipc blockade site (Figs 4c,d and 5).

**Figure 6:**
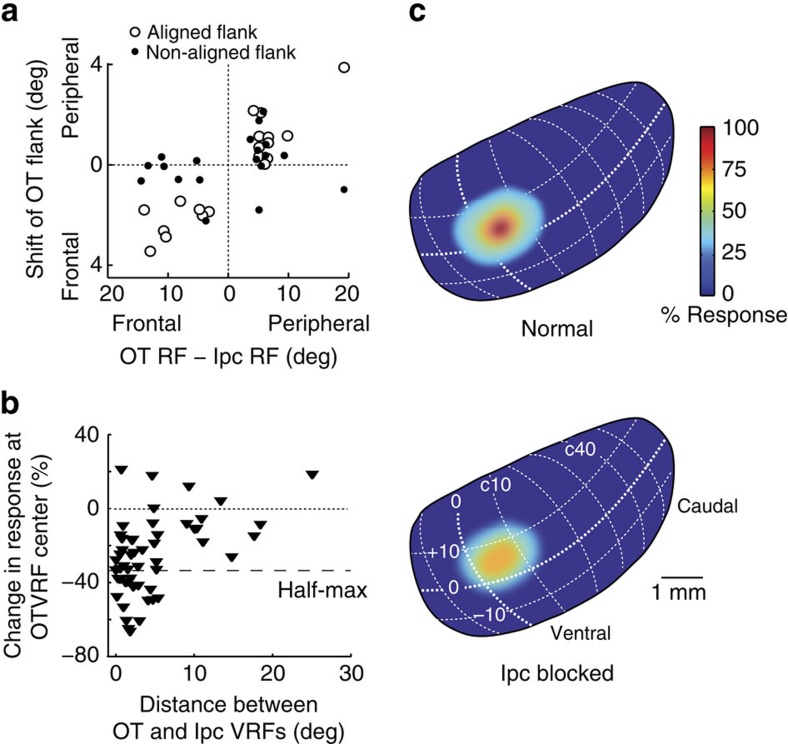
Effect of Ipc blockade on population visual responses in the OT. (**a**) Population summary of the effect of Ipc blockade on the Ipc-aligned half-max response (open circles) and the Ipc-non-aligned half-max response (solid dots) for non-aligned OT–Ipc sites (*n*=19). The directions of the shifts in half-max values are plotted as a function of the relative positions of the VRF centres for each OT–Ipc pair. (**b**) Extent of the blockade in the Ipc. The plot shows the effect of Ipc blockade on OT responses at the VRF centre. The data compare the strength of OT unit responses (*n*=52) to looming-dot stimuli (full contrast; 8° per s), measured at the centre of the OT VRF, before versus during Ipc blockade. This plot includes data from two OT–Ipc pairs for which the effect of blockade on the OT VRF centre was measured, but for which the effects on the entire OT tuning curve was not measured. Per cent change is plotted as a function of the distance, in degrees of space, between the OT and Ipc VRF centres. Dashed line indicates 50% of the maximum observed blockade effect (69% response reduction). (**c**) Illustration of the average population visual response across the OT space map, measured relative to the maximum response before Ipc blockade (colour bar; Methods). The drawings represent a lateral view of the space map on the surface of the OT^39^. Top: before Ipc blockade (Ipc connected); bottom: during Ipc blockade (Ipc disconnected).

The magnitude of the effect was similar whether the Ipc-aligned flank was located on the frontal or on the peripheral edge of the OT VRF. To analyse data collected from both sides of the brain, we translated the locations of OT VRFs from degrees right or left relative to Ipc VRFs, into degrees frontal (more rostral in the OT) or peripheral (more caudal in the OT). Plotted in these coordinates, the magnitude of the aligned flank shift (Fig. 6a, open circles) increased with the magnitude of the OT–Ipc misalignment (*r*^2^=0.75; *P*<0.005; *n*=19; linear regression), to values of up to 4°, when the aligned flank was on either the frontal or peripheral edge of the OT VRF.

At the same time, changes in the non-aligned OT flank (Fig. 6a, solid dots) were less systematic and not dependent on OT–Ipc misalignment (*P*=0.26; *n*=19; linear regression). Nevertheless, there was a tendency for this flank to also shift away from the Ipc VRF (Fig. 6a, solid dots in upper right and lower left quadrants; *P*=0.03; Wilcoxon signed rank test), the kind of effect expected from a release from lateral inhibition in the OT. As a consequence, both OT VRF flanks shifted away from the location represented at the Ipc blockade site.

For 8 of the 19 non-aligned pairs, Ipc blockade eliminated completely OT responses to the visual stimulus when the stimulus was located near the edge of the VRF on the aligned flank (Fig. 5a, asterisks). For all 8 of these pairs, Ipc blockade eliminated responses (response rate was not different from baseline activity; *P*>0.05; ANOVA) for at least one aligned-flank location, and for 4 of them, blockade eliminated responses for two of the sampled locations. For these locations, the modulation index reached its maximum value of 1 (Fig. 5b). These results show that Ipc input caused these OT units to become responsive to stimuli at locations that were beyond the edges of their VRFs measured without Ipc input (for example, Fig. 5a, black curve). These data suggest that Ipc input enlarges the representation of a stimulus in the OT by recruiting additional units that are located at and just beyond the edge of the OT population representation without Ipc input (Fig. 6c, increased size of the light blue area in top versus bottom representation).

The spatial precision of the effects of Ipc blockade on OT responsiveness (Fig. 6c) combines both the spatial extent of the blockade in the Ipc space map and the spatial precision of Ipc modulation of OT responses. Two lines of evidence indicate the spatial extent of the Ipc blockade. First, the magnitude of the change in responses at the OT VRF centre decreased with increasing OT–Ipc VRF separation (Fig. 6b). When the separation between the Ipc and OT VRFs exceeded 6°, the observed effect was always (*n*=11) <50% of the maximum observed effect (69% response reduction). Second, OT responses were modulated only by stimuli located near the VRF of the Ipc blockade site, and not by locations further away but still within their VRFs (Figs 2c, 4c and 5b). The boundary between the modulated and unmodulated locations within the OT VRFs reflected the extent of the blockade in the Ipc. Moreover, the differential modulation of responses within OT VRFs reflected the spatial precision of Ipc modulation in the OT: OT unit responses to stimuli located on the VRF left flank, in the VRF centre or on the right flank could each be modulated independently (sampled at intervals of 4°) by Ipc blockades with corresponding alignments (Figs 2, 4 and 5). These data suggest a spatial resolution for the Ipc's control of gain in the OT of ≤±4°. Of course, the true resolution available to the network (not confounded by the size of the Ipc blockade) is certainly much finer.

### Effects of the Ipc on OT gain and threshold

**Figure 7:**
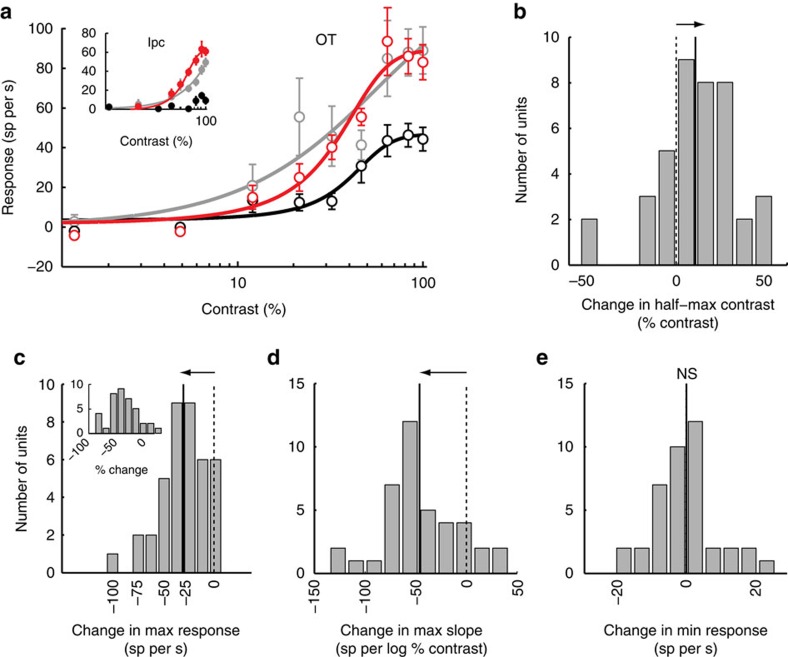
Effect of Ipc blockade on contrast-response functions in the OT for aligned OT–Ipc sites. (**a**) Data from a single OT–Ipc pair. The graph shows the responses (mean and s.e.m.) of an OT unit to various contrasts of a dark looming-dot stimulus (8° per s) before (red), during (black) and after (grey) iontophoretic application of kyn at the Ipc site. The inset shows the data recorded simultaneously from the Ipc injection site, and it uses the same colour code. Curves are best-fit sigmoidal functions (Methods). (**b**–**e**) Population summaries (*n*=40) of values derived from best-fit sigmoidal functions. Inset shows per cent changes. Dashed vertical line indicates zero change; solid vertical line indicates mean change; horizontal arrow indicates significant shift from zero (*P*<0.05; paired *t*-test). In **e**, the mean change was zero.

The decrease in OT unit responsiveness that resulted from blockade of visually driven input from the Ipc could have been due to a decrease in stimulus gain (increase in response threshold) and/or to a decrease in response gain^21^. To quantify the respective contributions of stimulus and response gains to the decrease in OT responsiveness, we measured (in a separate population of units; Methods) the effect of stimulus contrast on OT unit responses (contrast-response functions), without and with Ipc blockade, for stimuli centred in the OT VRF (Fig. 7a). A rightward shift of the contrast-response function would indicate a decrease in stimulus gain (an increase in threshold) and a vertical scaling of the function indicated a change in response gain (Methods).

Ipc blockade (Fig. 7a, inset) shifted the contrast-response function in the OT to the right (Fig. 7a, black), indicating an increase in unit threshold (half-max contrast; Methods) from 33 to 40%. Ipc blockade also decreased response gain, as indicated by the decrease in the maximum response from 94 to 45 sp per s, and the decrease in the maximum slope from 164 to 98 sp per log %contrast. Minimum responses remained unchanged. For this OT–Ipc pair, 12% of the variance caused by Ipc blockade was accounted for by a change in stimulus gain alone (horizontal shift of the function), and 96% of the variance was accounted for by a change in response gain alone (vertical scaling of the function).

Similar results were obtained from a population of 40 OT–Ipc pairs of sites with mutually aligned VRFs (OT–Ipc VRF alignment ≤4°). For this population, OT thresholds increased by an average of 9.2% contrast±2.2 (mean pre-blockade=29.6% contrast±1.8; mean during blockade=38.8% contrast±3; *P*<0.001; paired *t*-test; Fig. 7b). Maximum responses decreased by an average of 35% (mean pre-blockade=87 sp per s±14; mean during blockade=57 sp per s±9; *P*<0.001; paired *t*-test; Fig. 7c), and maximum slopes decreased by 38% (mean pre-blockade=121 sp per log %contrast±9.6; mean during blockade 75 sp per log %contrast±8.6; *P*<0.001; paired *t*-test; Fig. 7d). There was no consistent effect on minimum responses (mean pre-blockade=−5.2 sp per s±1.2; mean during blockade=−5.0 sp per s±1.2; *P*=0.89; paired *t*-test; Fig. 7e).

The contrast–response functions of all 40 OT units were fit well by a sigmoidal function (Methods), which accounted for an average of 97%±1 of the variance of the data. For 19 of the units, an average of 56%±5 of the variance in the contrast-response function measured during Ipc blockade was accounted for by a horizontal shift of the pre-blockade contrast-response function (change in stimulus gain); for the other 21 units, a horizontal shift of the pre-blockade function could not fit the data using the *nlinfit* MATLAB function. For all 40 units, an average of 92%±1 of the variance was accounted for by vertically scaling the pre-blockade functions (change in response gain); an additional 2% of the variance was accounted for by a subsequent horizontal shift of the functions. Thus, although the predominant effect of Ipc input was to increase response output gain in the OT, the evidence suggests that it also increased stimulus input gain.

### Effects of Ipc blockade on response time course and latency

**Figure 8:**
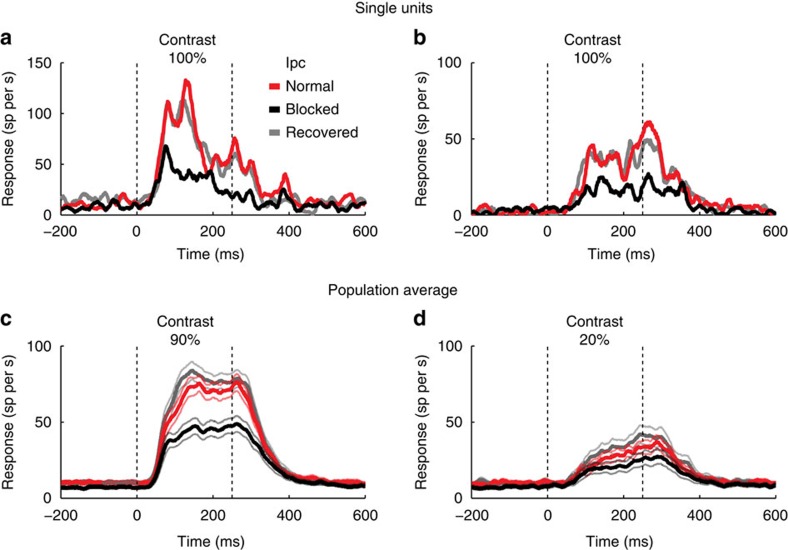
Effect of Ipc blockade on OT response time course. The data compare the time courses of OT unit responses to looming-dot stimuli (8° per s) presented at the centre of the VRF measured before, during and after Ipc blockade. Vertical dashed lines: duration of the stimulus. (**a**,**b**) Time courses of single OT units to full contrast stimuli. (**c**,**d**) Population average (*n*=41) time courses of responses to 90% contrast (**c**) and 20% contrast (**d**) looming dots. Shaded lines: s.e.m.

Previous research has demonstrated that cholinergic modulation of sensory responses in the visual cortex of monkeys engaged in an attention-demanding task persists throughout the duration of visual unit responses^22^. Analogously, we found that visual responses in the OT were modulated persistently by Ipc input. Some OT units exhibited stronger onset than sustained responses (Fig. 8a), while others responded with similar strength throughout the duration of the stimulus (Fig. 8b). In both cases, Ipc input enhanced approximately equally early and late responses, and weak and strong responses (Fig. 8c,d), throughout the duration of the response. In addition, we found that Ipc input also decreased the average response latency by 7 ms (from 63 to 56 ms; *P*=0.01; paired *t*-test, *n*=40; Methods).

## Discussion

This study reveals the contribution that the Ipc makes to the computation of the highest-priority location for attention and gaze. Although this cholinergic circuit exists in all vertebrate species, its role in information processing has only been studied in birds^23^. In birds, the Ipc has been shown to receive multimodal sensory information indicating the strength of sensory stimuli across space, as well as top-down information from the forebrain gaze field^9^,^24^,^25^. The Ipc also receives strong inhibition from the nucleus isthmi pars magnocellularis (Imc; analogous to the periparabigeminal lateral tegmental nucleus in mammals), a GABAergic nucleus that is responsible for mediating global competitive inhibition in the network^26^. The Imc represents the ongoing evaluation of the strength of activation at each location in the space map relative to the strongest activation anywhere in the map, and it suppresses responses at all but the strongest location. The strength of activation in the OT space map depends critically on the gain provided by the Ipc circuit^27^. As demonstrated by this study, Ipc input amplifies and improves the spatial and contrast resolution of OT responses, and shifts OT VRFs towards the location represented by the Ipc activity. By enhancing the OT population response in a location-specific manner, the Ipc controls the network's spatial bias in the computation of the highest-priority location.

The results of this study establish causal links between a particular neural circuit, dynamic VRF shifts and gain control. The effects of the Ipc on OT responses involve the regulation of both response (output) gain and stimulus (input) gain. The regulation of stimulus gain (indicated by changes in response thresholds) could be mediated by cholinergic presynaptic facilitation of glutamate release from afferent terminals^28^,^29^, a mechanism that also operates in the mammalian neocortex^30^. A mechanism for regulating output gain (indicated by changes in maximum responses and slopes of contrast-response functions) has not been demonstrated in the OT. However, acetylcholine release in the OT has been shown to directly drive a special population of local inhibitory neurons that, in turn, inhibit other local inhibitory neurons^31^. Such an acetylcholine-driven, ‘inhibition of inhibition' motif has been shown to control the output gain of pyramidal neurons in the mammalian sensory neocortex^32^,^33^.

The capacity of the Ipc to modulate responses in the OT is spatially precise and large in magnitude. The Ipc is capable of modulating gain selectively within subregions of OT VRFs, consistent with the exquisite anatomical precision of the Ipc–OT projection^11^ (Fig. 1c). The Ipc's influence on gain is powerful: our average blockade, which decreased Ipc activity by 54%, decreased the responses of OT units with aligned VRFs by an average of 31%. For neurons near the boundaries of the stimulus representation, the modulation of OT responses was even greater: Ipc blockade either eliminated responses completely or caused exceptionally large modulations. Moreover, additional Ipc activation caused by descending forebrain pathways, delivered either directly to the Ipc or indirectly via OT neurons^20^, is likely to increase the modulatory effects of the Ipc even beyond the values reported in this study.

The effects of Ipc input, including both the space-specific enhancements of OT responses and the broadcasting of a synchronizing rhythm across the OT layers^13^, are accomplished without directly driving OT output neurons^31^. Instead, Ipc activity shifts OT circuitry towards a high-gain state capable of producing strong, rhythmic output. Therefore, when activated by top-down signals from the forebrain^9^, space-specific Ipc input to the OT would put the midbrain network into a high gain, high-resolution state for a specific location without evoking phosphenes.

Dynamic shifts of sensory receptive fields have been reported in the owl OT in response to electrical microstimulation in the forebrain gaze field^19^. Such microstimulation, applied at the representation of a particular location, increases the responsiveness of OT neurons in the deeper layers at the corresponding location, but does not, by itself, evoke spike activity from those neurons^34^. At the same time, it causes a decrease in the output gain of OT neurons at non-corresponding locations, resulting presumably from the suppression of Ipc activity at those locations by the globally inhibitory Imc^26^. As a consequence, the receptive fields of OT neurons shift towards the location represented by the descending forebrain signal. These effects mimic the Ipc influences on OT VRFs that we report in this study.

Dynamic shifts of VRFs have also been reported in the superior colliculus and neocortex of monkeys engaged in goal-directed saccade tasks^35^,^36^. Immediately before a monkey initiates an eye saccade towards a particular location, visual neurons in the superior colliculus and neocortex increase their responsiveness to visual stimuli located near the goal of the planned saccade. As a result, the VRFs of these neurons shift dynamically towards the goal of the impending saccade. Although these effects are reminiscent of the space-specific modulations of visual responsiveness reported in this study, their magnitude suggests that they are mediated by a different mechanism.

The midbrain stimulus selection network computes a categorical representation of the location of the highest-priority stimulus based on a global, competitive evaluation of the relative strength of neural activity across the OT space map^37^. The Ipc circuit modulates powerfully the strength of responses in the OT space map in a highly space-specific manner. Thus, the level of Ipc activity at each location in the OT space map controls the capacity of that location to compete, via the Imc circuit, for the designation of ‘highest-priority location'. This capacity will regulate the balance of bottom-up stimulus competition in the midbrain network, and it provides a mechanism by which descending forebrain signals can bias the competition to favour the selection of a particular stimulus or location as the next target for attention and gaze. Given that this network appears early and remains throughout vertebrate evolution^23^, we propose that the analogous midbrain circuits perform the same function in all vertebrates, including humans.

## Methods

### Animals

Experiments were performed on 12 head-fixed, non-anaesthetized, adult barn owls (*Tyto alba)*. Both male and female birds were used. All procedures for bird care and use were approved by the Stanford University Institutional Animal Care and Use Committee, and were in accordance with the US National Institutes of Health and the Society for Neuroscience guidelines for the care and use of laboratory animals.

Measurements were made both in tranquilized and in non-tranquilized owls. At the start of each experiment, a mixture of isofluorane (1.5%) and nitrous oxide/oxygen (45:55) was administered to anaesthetize the bird while it was positioned and secured in the experimental rig. Isofluorane was then turned off and was not turned back on for the remainder of the experiment. In initial experiments, the owls remained tranquilized with the nitrous oxide/oxygen mixture throughout the experiment. In later experiments, the tranquilizing mixture was discontinued after the owl was positioned in the chamber. The results were not different under these two conditions, and the data have been combined.

The owls were suspended in a tube in a darkened sound chamber. The head was bolted in place so that the visual axes aligned with 0°az, 0°el of a calibrated tangent screen located 30 cm in front of the bird; the eyes of adult barn owls are stationary (±2°) in the head.

### Neurophysiology

First, a multi-barrelled, iontophoretic/recording electrode was positioned at a desired site in the Ipc, and the VRF at that site was measured. Second, a tungsten electrode (A-M Systems; epoxy-coated; 2–5 MΩ at 1 kHz) was used to isolate single units in layers 11–13 of the OT that exhibited a VRF that was either aligned, or non-aligned by a specific amount, with the VRF at the Ipc injection site.

### Iontophoretic blockade

Excitatory drive to Ipc neurons was blocked focally and reversibly by iontophoretic application of kyn (Sigma, 40 mM, 8.5–9 pH), a broad-spectrum blocker of ionotropic glutamate receptors. Data were included in the analysis only when unit responses recovered to within 75% of pre-drug levels within 20 min following cessation of drug application (Fig. 2a, grey). The drug was delivered through a multi-barreled electrode (FHC, three-barrel borosilicate capillary tubing, 1.2 or 1.5 mm outer diameter for each barrel; tip diameter=25–30 μm for all three barrels together) in the Ipc that allowed us to apply the drug and, at the same time, to record multi-unit activity documenting the effectiveness of the drug at the injection site. Kyn was ejected by passing −500 nA through the drug barrel with a DAGAN 6400Adv iontophoresis amplifier. The recording barrel contained a carbon fibre and was saline filled. The third barrel was also saline-filled and was used to balance the charge delivered by the kyn-containing barrel.

### Visual stimuli

Experiments were conducted in a darkened sound chamber. Spatial tuning and contrast-response functions were measured with looming dot stimuli, generated with Matlab software. Negative contrast (dark) dots expanded linearly in size for 250 ms, at a rate of 8.0° per s, starting from a size of 0.6° in radius; OT units habituate less to looming stimuli than to other kinds of stimuli. Details of these stimuli and their effects on OT responses have been discussed previously^38^. Stimulus location or contrast was varied in a randomly interleaved fashion, and each parameter value was repeated 12–20 times, with an inter-stimulus interval of 3 s.

### Data analysis and statistical tests

We analysed data only from OT–Ipc pairs of sites for which responses returned to within 75% of pre-block levels within 20 min of cessation of drug application. All analyses were carried out with custom Matlab code. Activity was recorded for 1,000 ms, beginning 250 ms before stimulus onset. Response rates were calculated by subtracting the spike rate during a baseline period 200 ms before stimulus onset from the spike rate during a 300 ms time window beginning 20 ms after stimulus onset. Spontaneous activity was calculated from the pre-stimulus activity averaged across all trials that tested spatial tuning.

Parametric or nonparametric paired statistical tests were applied on the basis of whether the distributions being compared were Gaussian or not (Lilliefors test of normality). For normally distributed data, values are given as mean±s.e.m.

The VRF for each unit was defined as the set of locations at which stimuli evoked responses above baseline. Responses were fit with a Gaussian function. VRF centre was the location at the centre of the Gaussian function; width at half-max was the width at the midpoint between the maximum and minimum values. The locations of VRF flanks were quantified as the locations of these midpoints.

Modulation index was calculated using values from the Gaussian functions, as follows: responses (pre-blockade−during blockade)/(pre-blockade+during blockade). Modulation index was calculated only when the pre-blockade response to a location was significantly greater than baseline rate (ANOVA; *P*<0.05). Negative responses during blockade were treated as zero response, which yielded modulation index=1.

Spatial discrimination index (*d*′) was calculated from the means and s.d.'s of responses to stimuli at different azimuthal locations across the VRF using the following formula:


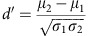


where *μ*_1_ and *μ*_2_ are the mean responses for data sets 1 and 2, respectively, and *σ*_1_ and *σ*_2_ are the s.d.'s of those data sets. Max *d′* was the largest *d′* value calculated from responses to any two locations at 6° intervals across the spatial tuning curve.

Representations of the effect of Ipc blockade on average population responses across the OT space map (Fig. 6c) were estimated based on the known topography of the OT space map in the barn owl^39^. Average population response was derived from a four-point running average of the responses without and with Ipc blockade for visual stimuli located at the centre of the Ipc VRF for each OT–Ipc pair of sites, as a function of the distance between the OT and Ipc VRF centres for each pair. Responses were quantified as the % of the maximum response measured before Ipc blockade. The representations assume radial spatial symmetry of the effect and an isotropic representation of space in the OT space map.

Contrast–response functions were measured by presenting stimuli at the VRF centre. These functions were measured in the same birds, but in experiments that were separate from those in which spatial tuning was measured. Contrast values from 0 to 100% were tested in a random, interleaved order, and responses were best-fit with the logistic function:


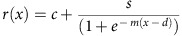


where *r*(*x*) is the response at contrast *x*, *c* is the minimum response (*y*-offset), *s* is the maximum response, *d* is the stimulus contrast that evoked 50% of the maximum response (half-max contrast) and *m* is the maximum slope.

The respective contributions of stimulus gain and response gain^21^ to the change in the contrast-response function caused by Ipc blockade, were quantified as follows: first, the contrast-response data measured before Ipc blockade were best-fit with the logistic function (pre-blockade function). To quantify the contribution of stimulus gain, the contrast-response data measured during Ipc blockade were best-fit holding all parameters of the pre-blockade function constant, leaving only the half-max value as a free parameter and calculating the goodness of fit (*R*^2^ value). To quantify the contribution of response gain, the contrast-response data measured during Ipc blockade were best-fit holding all parameters of the pre-blockade function constant, leaving only the max response value as a free parameter and calculating the goodness of fit (*R*^2^ value).

Response latency was measured as the time relative to the onset of a high-contrast looming dot when unit responses exceeded 2.58 × the s.d. of the resting discharge rate.

Peristimulus-time histograms were generated by counting spikes in 1 ms time bins, and smoothing the data with an 8 ms, sliding Gaussian filter.

### Data availability

The data that were collected for this study are available from the corresponding author on request.

## Additional information

**How to cite this article:** Asadollahi, A. and Knudsen, E. I. Spatially precise visual gain control mediated by a cholinergic circuit in the midbrain attention network. *Nat. Commun.* **7,** 13472 doi: 10.1038/ncomms13472 (2016).

**Publisher's note:** Springer Nature remains neutral with regard to jurisdictional claims in published maps and institutional affiliations.
